# Identification of *Pyrus* Single Nucleotide Polymorphisms (SNPs) and Evaluation for Genetic Mapping in European Pear and Interspecific *Pyrus* Hybrids

**DOI:** 10.1371/journal.pone.0077022

**Published:** 2013-10-14

**Authors:** Sara Montanari, Munazza Saeed, Mareike Knäbel, YoonKyeong Kim, Michela Troggio, Mickael Malnoy, Riccardo Velasco, Paolo Fontana, KyungHo Won, Charles-Eric Durel, Laure Perchepied, Robert Schaffer, Claudia Wiedow, Vincent Bus, Lester Brewer, Susan E. Gardiner, Ross N. Crowhurst, David Chagné

**Affiliations:** 1 Istituto Agrario San Michele all’Adige Research and Innovation Centre, Foundation Edmund Mach, San Michele all’Adige, Trento, Italy; 2 The New Zealand Institute for Plant & Food Research Limited (Plant & Food Research), Palmerston North Research Centre, Palmerston North, New Zealand; 3 Institut National de la Recherche Agronomique (INRA), UMR1345 Institut de Recherche en Horticulture et Semences, SFR 4207 Quasav, Pres L’UNAM, F-49071 Beaucouzé, France; 4 Université d’Angers, UMR1345 Institut de Recherche en Horticulture et Semences, F-49045 Angers, France; 5 AgroCampus-Ouest, UMR1345 Institut de Recherche en Horticulture et Semences, F-49045 Angers, France; 6 Institute of Food Nutrition and Human Health, Massey University, Palmerston North, New Zealand; 7 School of Biological Sciences, University of Auckland, Auckland, New Zealand; 8 National Institute of Horticultural and Herbal Science, Pear Research Station, Naju, Republic of Korea; 9 Plant & Food Research, Mount Albert Research Centre, Auckland, New Zealand; 10 Plant & Food Research, Hawke’s Bay Research Centre, Havelock North, New Zealand; 11 Plant & Food Research, Motueka Research Centre, Motueka, New Zealand; Virginia Tech, United States of America

## Abstract

We have used new generation sequencing (NGS) technologies to identify single nucleotide polymorphism (SNP) markers from three European pear (*Pyrus communis* L.) cultivars and subsequently developed a subset of 1096 pear SNPs into high throughput markers by combining them with the set of 7692 apple SNPs on the IRSC apple Infinium® II 8K array. We then evaluated this apple and pear Infinium® II 9K SNP array for large-scale genotyping in pear across several species, using both pear and apple SNPs. The segregating populations employed for array validation included a segregating population of European pear (‘Old Home’×‘Louise Bon Jersey’) and four interspecific breeding families derived from Asian (*P. pyrifolia* Nakai and *P. bretschneideri* Rehd.) and European pear pedigrees. In total, we mapped 857 polymorphic pear markers to construct the first SNP-based genetic maps for pear, comprising 78% of the total pear SNPs included in the array. In addition, 1031 SNP markers derived from apple (13% of the total apple SNPs included in the array) were polymorphic and were mapped in one or more of the pear populations. These results are the first to demonstrate SNP transferability across the genera *Malus* and *Pyrus*. Our construction of high density SNP-based and gene-based genetic maps in pear represents an important step towards the identification of chromosomal regions associated with a range of horticultural characters, such as pest and disease resistance, orchard yield and fruit quality.

## Introduction

One of the biggest challenges for plant biologists has long been to associate genetic variations with phenotypic traits. The recent technological revolution initiated by new generation sequencing (NGS) has enabled the sequencing of the entire genome of complex organisms, including the higher plants grape [1], [2], maize [3], peach [4], apple [5], potato [6], tomato [7] and most recently, Chinese pear [8]. NGS also enables the inventory of entire sets of DNA variations in genomes, through the re-sequencing of multiple accessions of the same species and alignment of these sequences to the reference genome, for the purpose of *in silico* detection of DNA polymorphisms [9]–[16].

Single nucleotide polymorphisms (SNPs) are single base variations in DNA sequences that are abundant in plant genomes and are useful for identifying differences within individuals or populations as well as identifying genetic loci associated with phenotypic variation. Within coding regions, SNPs may be defined as non-synonymous or synonymous (resulting in an amino acid change or not) and are also found in gene-regulating regions (e.g. in promoters, untranslated mRNA regions and introns). Once polymorphisms have been detected by NGS, the next challenge is to screen large genetic populations with multiple markers simultaneously. While re-sequencing can be used for both SNP discovery and genotyping of the entire set of polymorphisms of a species [17], high throughput SNP arrays, such as the Infinium® II assay (Illumina Inc.), are effective technologies for genotyping of large populations.

High throughput SNP arrays have been recently developed for a range of fruit tree species. In Rosaceae, an apple SNP array was developed by the International RosBREED SNP consortium (IRSC) (www.rosbreed.org) [9]. This 8K SNP array v1 contains 7867 SNPs, of which 5554 proved to be genome-wide polymorphic SNPs in apple. The International Peach SNP Consortium (IPSC) developed a 9K SNP array for peach that includes 8144 SNPs, 84.3% of which exhibit polymorphism when screened over 709 accessions of peach (comprising peach cultivars, wild related *Prunus* species and interspecific hybrids) [10]. IRSC also led the development of a 6K SNP array for cherry, with 1825 verified polymorphic SNPs in sweet cherry and 2058 in sour cherry [18]. In *Citrus*, 54 accessions and 52 interspecific hybrids between pummelo and Clementine were genotyped using a 1457 GoldenGate® SNPs assay developed from clementine BAC-end sequencing. Out of 622 SNPs showing consistent results, 80.5% were demonstrated to be transferable to the whole *Citrus* gene pool [19].

The genus *Pyrus* includes both European (*Pyrus communis*) and Asian pears (*P. pyrifolia* or Japanese pear, and *P. bretschneideri*, commonly known as Chinese pear). To date, only a few genetic maps have been developed for *Pyrus* and none of these contains SNP markers. The first map was constructed using random amplified polymorphic DNA (RAPD) markers in a *P. pyrifolia* cross between ‘Kinchaku’ and ‘Kosui’ [20]. Yamamoto et al. [21], [22] developed the second generation of pear maps based on amplified fragment length polymorphism (AFLPs) and transferrable apple and pear simple sequence repeat (SSRs), using an interspecific cross between ‘Bartlett’ (*P. communis*) and ‘Hosui’ (*P. pyrifolia*). As the ‘Bartlett’×‘Hosui’ map contained SSRs derived from both pear and apple, this study enabled the assessment of genome synteny between pear and apple and suggested that these species have co-linear genomes. Apple and pear markers had also been used earlier to generate maps for the two European pear cultivars ‘Passe Crassane’ and ‘Harrow Sweet’ [23]. SSR markers developed from both apple and pear were also used by Celton et al. [24] to build an integrated map of the *P. communis* cultivars ‘Bartlett’ and ‘La France’, along with two apple rootstocks. Lu et al. [25] screened the interspecific pear population ‘Mishirazi’ (*P. pyrifolia*×*P. communis*)×‘Jinhua’ (*P. bretschneideri*) with apple SSRs and were able to construct a genetic map. However, the number of markers used in all these studies was limited to few hundreds. Recently, NGS was used to develop a genetic map of ‘Bayuehong’ (*P. bretschneideri*×*P. communis*)×‘Dangshansuli’ (*P. bretschneideri*) to anchor the Chinese pear genome; however, these SNPs were not evaluated for the screening of large segregating populations [8].

In this study, we used NGS to detect SNPs in the pear genome, to enable the design of a medium throughput SNP assay. These new pear SNPs were evaluated for genetic map construction using five segregating populations of European and Asian pear origin. Our incorporation of the new pear SNPs into the IRSC apple Infinium® II 8 K array [9], enabled the study of SNP transferability not only within the genus *Pyrus*, but also between the genera *Malus* and *Pyrus*.

## Materials and Methods

### NGS Sequencing of Pear Cultivars

A SNP detection panel consisting of three European pear (*P. communis*) cultivars was chosen for low coverage whole-genome sequencing. The individuals were ‘Bartlett’ (a.k.a. ‘Williams Bon Chrétien’), ‘Old Home’ (OH) and ‘Louise Bon Jersey’ (LBJ). These accessions were chosen as ‘Bartlett’ is a founder of most breeding programmes worldwide, and OH and LBJ are the parents of a segregating population developed at Plant & Food Research (PFR). Each accession was sequenced using one lane of Illumina GA II with 75 cycles per read and small insert paired-end sequencing, as described in [9].

Two pear unnormalized cDNA libraries were prepared by vertis Biotechnologie AG for the European pear cultivar ‘Max Red Bartlett’ following VERTIS customized protocol (http://www.vertis-biotech.com/). One run of 454 sequencing on a Roche/454 GS FLX Sequencer was performed.

### Bioinformatics Detection and Selection of SNPs for Array

A *de novo* assembly was performed for the ‘Bartlett’ sequencing data using AbySS 1.2.1 (k = 43). Contigs of 600 bp or larger were used as a reference genome set. The sequencing data from OH and LBJ were mapped to the reference genome set of ‘Bartlett’ using *Soap2.20* (-p 8 -M 4 -v 5 -c 52 -s 12 -n 5 -r 2 -m 50 -x 600). *Soap* output files were split into a single file per contig and each contig file sorted by location of the mapped reads. *SoapSNP* was used for SNP detection and filtering with the same parameters as described in [9]. The detected SNPs were then subjected to filtering, where calls were discarded when the quality score was less than 20; fewer than two reads per genotype were present; overall coverage depth was greater than the average coverage plus three standard deviations; the site was at least 25 bases away from another SNP call; and the SNPs were not located within regions associated with a set of candidate genes. The candidate gene set used for filtering consisted of 2559 transcription factor sequences from *Malus*×*domestica* [5]. Locations within pear were defined by mapping these sequences to the reference genome set of ‘Bartlett’ using *gmap* with command line options -K 3000–L 50000.

454 cDNA reads were assembled using CAP3 [26]. Contigs were aligned to the reference *M.*×*domestica* genome and only unique alignments were considered to avoid parology issues. SNPs were predicted using a customized bioinformatics pipeline and selected to be well spread over the 17 apple chromosomes.

The Illumina Infinium® assay design tool (ADT) was used on the detected SNPs with a threshold of 0.7. These pear SNPs were synthesized as probes and located on the same array as the IRSC apple Infinium® II 8 K array [9].

### Plant Material for SNP Array Evaluation

**Figure 1:**
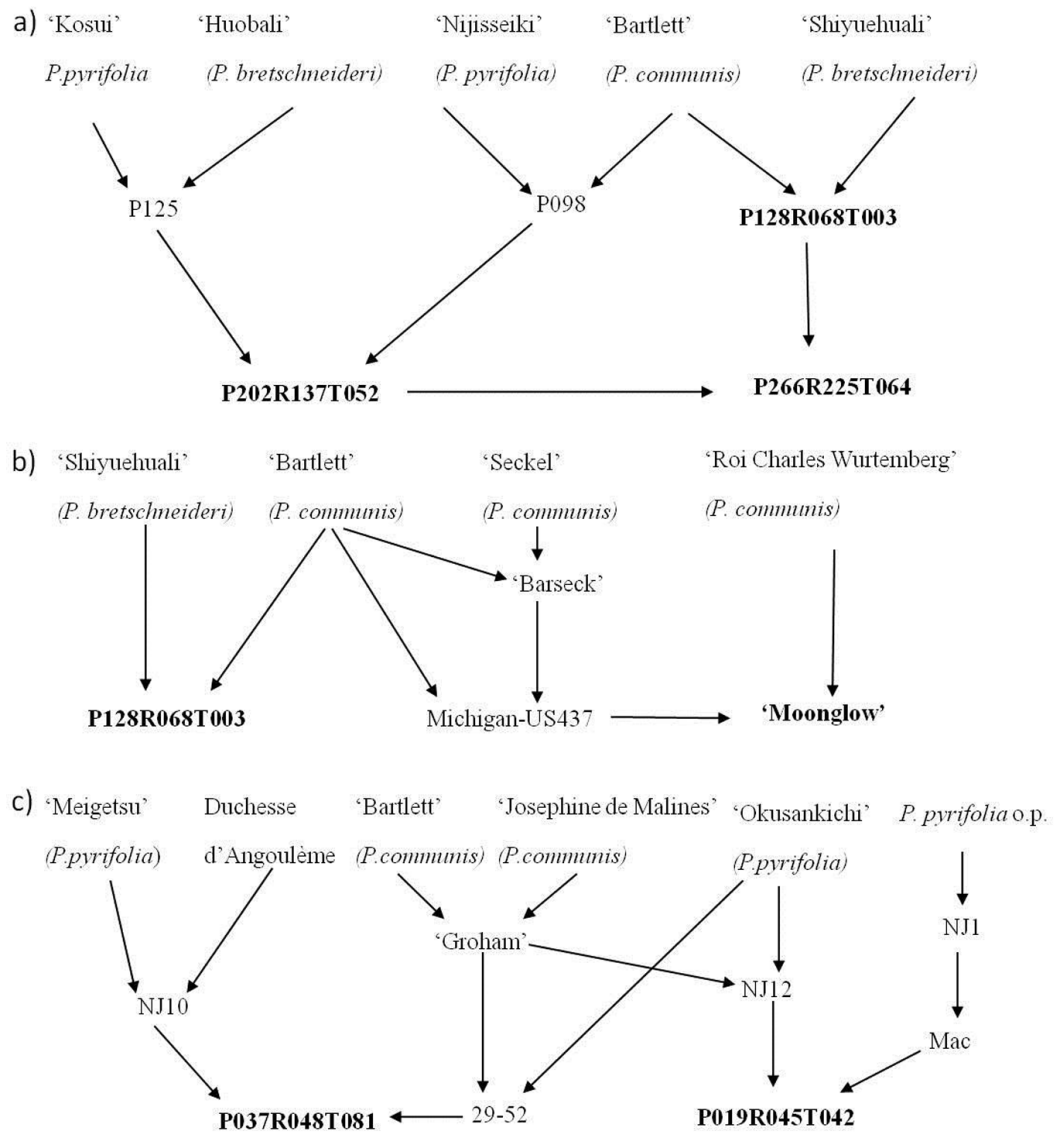
Pedigree diagrams for segregating populations used for SNP evaluation. A) P128R068T003×‘Moonglow’; B) P037R048T081×P019R045T042, and C) P202R137T052×P128R068T003 and P202R137T052×P266R225T064.

Five pear segregating populations were screened using the apple and pear Infinium® II 9K SNP array. No permission was required to collect plant material and pear is not an endangered or protected species. These were one *P. communis* intraspecific family and four interspecific (*P. bretschneideri*, *P. communis* and *P. pyrifolia*) pear populations: OH×LBJ, of 297 F1 individuals and both parents; P128R068T003×‘Moonglow’ (T003×M), of 220 F1 individuals and both parents; P019R045T042×P037R048T081 (T042×T081), of 142 F1 individuals and both parents; P202R137T052×P128R068T003 (T052×T003), of 91 F1 individuals and T003 parent only; and P202R137T052×P266R225T064 (T052×T065), of 123 F1 individuals and T064 parent only, since parent T052 has been lost. Figure 1 shows the relationships among the interspecific populations. The interspecific hybrid populations were developed as part of the PFR pear breeding programme [27]. Half the P128R068T003×‘Moonglow’ population was grown at INRA, Angers (France) and genotyped at the Fondazione Edmund Mach (FEM, Italy), and the other half was grown at PFR, Motueka and genotyped at AgResearch Limited, Invermay in New Zealand, together with the other four populations. DNA extraction of OH×LBJ, T042×T081 and T052×T003 populations was performed using a CTAB extraction method [28], followed by purification with NucleoSpin® columns (Macherey-Nagel GmbH & Co. KG). DNA from the T003×M and T052×T064 populations was extracted using the QIAGEN DNeasy Plant Kit (QIAGEN GmbH, Hilden, Germany). DNA quantifications were carried out using a NanoDrop™ 2000c spectrophotometer (Thermo Fisher Scientific Inc.).

### SNP Genotyping and Data Analysis

**Figure 2:**
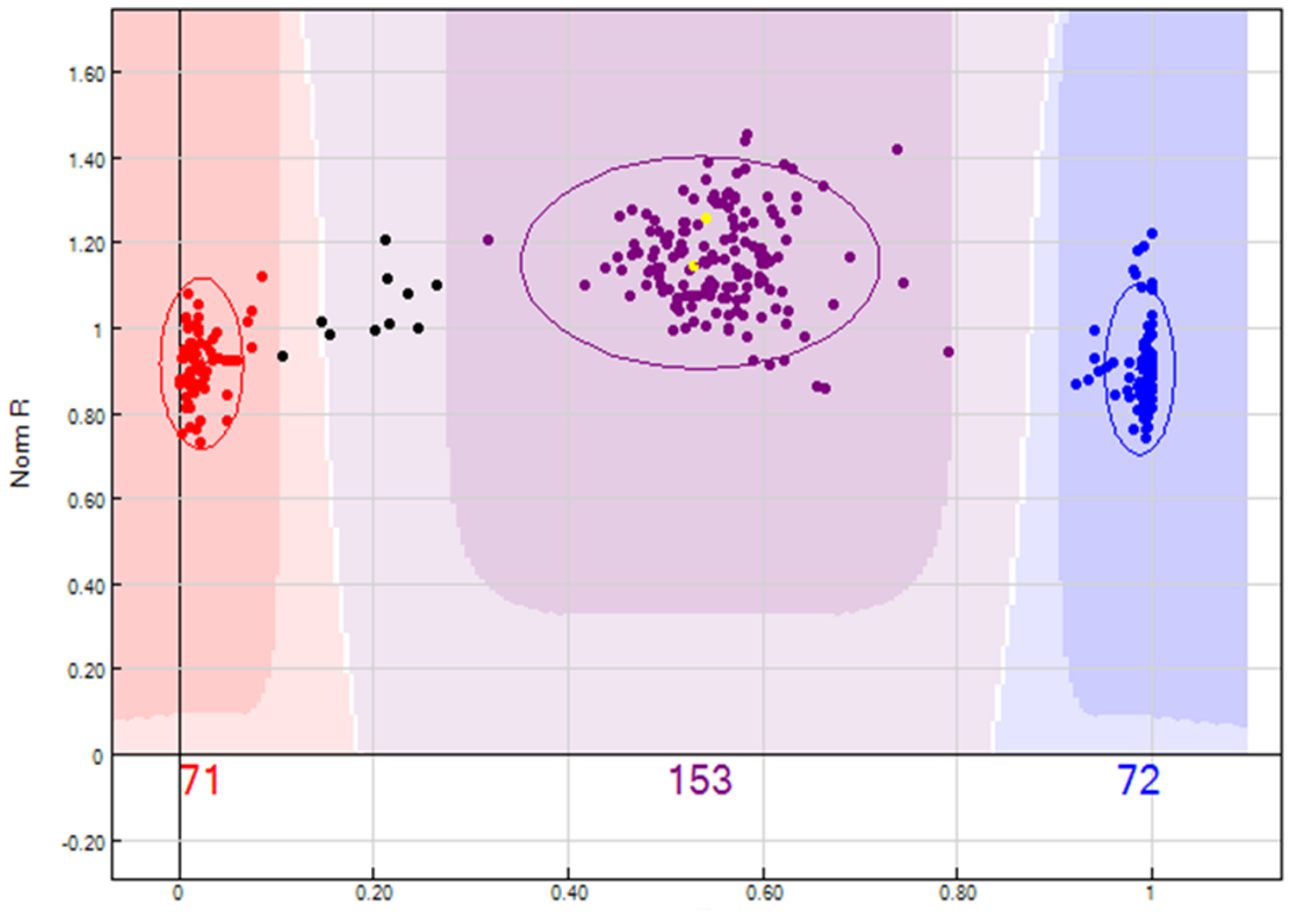
A typical example of an AB×AB SNP (ss527787957), as represented in GenomeStudio. Parents ‘Old Home’ and ‘Louise Bon Jersey’ are indicated in yellow; the red cluster is identified as AA, the blue as BB and the purple as AB genotype. The total number of the individuals analyzed here is 297 and the segregation ratio is 1∶2:1.

Genomic DNA was amplified and hybridized to the apple and pear Infinium® II 9K SNP array following the Infinium® HD Assay Ultra protocol (Illumina Inc., San Diego, USA) and scanned with the Illumina HiScan. Data were analyzed using Illumina’s GenomeStudio v 1.0 software Genotyping Module, setting a *GenCall* Threshold of 0.15. The software automatically determines the cluster positions of the AA/AB/BB genotypes for each SNP and displays them in normalized graphs (Figure 2). A systematic method was used to evaluate the SNP array data employing quality metrics from GenomeStudio (Illumina): GenTrain score ≥0.50, minor allelic frequency (MAF) ≥0.15 and call rate >80%. A Chi-square test at a significance of 0.01 was performed to determine distortion of markers from the expected segregation. SNPs that were highly distorted or which had the genotype of one or both parents missing were manually edited in GenomeStudio. The SNPs for which 25% or 50% of the individuals were not called in clusters were manually edited, since this kind of segregation may have been due to SNPs with null alleles.

### Simple Sequence Repeat Genotyping

The T003×M population was genotyped with apple and pear microsatellite markers as well as SNPs. Fifty-four SSRs were selected based on the ‘Bartlett’ consensus map developed by Celton et al. [24] and one SSR, *Md-Exp 7*, from the work of Costa et al. [29]. They were first screened for polymorphism over DNA extracted from both parents and five individuals of the progeny, and then screened over the subset of the T003×M population raised at INRA (Table S1). PCR amplifications were performed in a final volume of 12.5 uL containing 10 ng of genomic DNA, 1x buffer, 2 mM MgCl_2_, 0.2 mM of each dNTP, 0.4 uM of each forward and reverse primer and 0.75 U of AmpliTaq Gold® DNA polymerase (Applied Biosystems® by Life Technologies™). All SSR amplifications were performed in a Biometra T gradient Thermocycler (Biometra GmbH, Göttingen, Germany) or in a Bio-Rad C-1000 thermocycler (Bio-Rad Laboratories, Hercules, CA) at FEM (Italy) and INRA, Angers (France) under the following conditions: an initial denaturation at 95°C for 5 min, followed by 36 cycles of 95°C for 30 sec, TA (an optimal annealing temperature for each primer was used) for 30 sec, 72°C for 1 min, finishing with a final extension at 72°C for 7 min. Fragment analysis was performed with an ABI PRISM_3730 capillary sequencer (Applied Biosystems® by Life Technologies™) in a final mix of 0.5 uL of PCR product, 9.97 uL formamide and 0.03 uL of 500-LIZ dye, denaturated for 3 min at 95°C. Fragment sizing was performed with GeneMapper software v. 4.0 (Applied Biosystems® by Life Technologies™).

### Linkage Mapping Analysis

The genetic maps of both parents of all five populations were constructed using JoinMap v3.0 and v4.0 software [30], based on the SNP data for each individual population, except for the T003×M population, where both the SNP and SSR data were used. Linkage groups were determined with a LOD score of 5 and higher for grouping and the Kosambi function was used for map calculation. The maps were drawn and aligned using MapChart v2.2 [31].

### Pear SNP Alignment to the Apple Genome Sequence

The pear SNPs included in the array were aligned to the apple genome assembly [5] using BLASTN analysis of the SNP flanking sequence against the ‘Golden Delicious’ (GD) genome assembly. A BLASTN cutoff of an alignment length >100 nucleotides and an e-value<e-30 were used.

## Results

### SNP Detection and Selection for 1 K Pear Array

In total, 34,082,435, 35,687,533 and 25,167,853 paired-end reads were generated for ‘Bartlett’, OH and LBJ, respectively. The *de novo* assembly genome set of ‘Bartlett’ consisted of 78,748 contigs of 600 bp or greater in length containing a total of 79,067,993 bases, with a maximum contig length of 15,094 bases, N50 of 1004 bases, N90 of 658 bases, and an average contig length of 1004 bases. A total of 73,214 SNPs were predicted by SoapSNP when reads of OH and LBJ were aligned to the genome of ‘Bartlett’ using the Soap aligner, corresponding to one SNP per 1079 bases. In total, 1,456 SNPs passed the filtering criteria and were then subjected to the Illumina ADT. This yielded 1107 SNPs, of which 1064 were included in the final SNP array.

A total of 144,816 high quality 454 sequence reads were generated. Total sequence output was 32,418,987 bases, with an average read length of 224 bases. Quality filtered sequences were *de novo* assembled using CAP3. The average depth of assembly for all samples was ∼2.5. A total of 1751 cDNA SNPs were predicted using a customized bioinformatics pipeline and 69 experimentally validated by M. Troggio (unpublished data) that passed the Illumina ADT design, were selected for inclusion in the SNP array.

In total, 1133 pear SNPs were incorporated in the final array, making a grand total of 9000 attempted apple and pear SNPs (Table S2).

### SNP Chip Evaluation

Of the 1133 attempted pear SNPs, 1096 (96.7%) were successful bead types on the IRSC Infinium® II (Illumina Inc.) array. When the 1096 pear and 7692 apple bead types were evaluated using five segregating populations, twelve and three individuals from the T003×M and T052×T003 populations, respectively, did not hybridize well to the BeadChip and were excluded from the clustering, which resulted in 873 F1 individuals that were used for evaluating the SNP array. All the 1096 pear SNPs hybridized well, resulting to be either polymorphic or monomorphic in at least one population. Of the apple SNPs, 7562 out of the total 7692 bead typed (98.3%) were either polymorphic or monomorphic in at least one population, while only 130 showed low quality hybridization. All 1096 pear SNPs hybridized pear DNA and were either monmorphic or polymorphic.

In total, 1528 unique pear and apple-derived SNPs (872 pear SNPs and 656 apple SNPs) were polymorphic in at least one segregating population, with 713, 508, 437, 442 and 711 polymorphic SNPs for the OH×LBJ, T003×M, T042×T081, T052×T003 and T052×T064 populations, respectively (Table 1). For the newly developed pear SNPs, the polymorphism rate was variable and depended on the informative parent. *P. communis* parents had higher polymorphism rate (from 25.9% to 35.1%, for ‘Moonglow’, OH and LBJ) than Asian×European hybrid parents (from 2.9% to 21.4%, for T003 and T064, respectively). The number of polymorphic apple SNPs per pear population ranged from 115 to 381 out of 7692 beadtypes (1.5 to 5.0% polymorphic SNPs per population). When the transfer rate of the new pear SNPs was evaluated in the apple ‘Royal Gala’×‘Granny Smith’ segregating population, it was similar to the apple SNP to pear transfer rate, with 13 (1.2%) polymorphic pear SNPs.

**Table 1:** Number of polymorphic and mapped apple and pear markers for each segregating population.

			Polymorphic markers			Mapped markers		
	Population	Marker segregation	Pear SNPs	Apple SNPs	Total	Pear SNPs	Apple SNPs	Total
***Pyrus***	**OH×LBJ (n = 297)**	ABxAA/BB	213	50	263	194	41	235
		ABxAB	128	9	137	123	9	132
		BB/AAxAB	257	56	313	229	49	278
		total	598	115	713	546	99	645
	**T003**×**M (n = 220)**	ABxAA/BB	21	113	134	16	105	121
		ABxAB	11	4	15	11	3	14
		BB/AAxAB	273	86	359	271	77	348
		total	305	203	508	298	185	483
	**T042**×**T081 (n = 142)**	ABxAA/BB	146	47	193	140	42	182
		ABxAB	23	3	26	23	3	26
		BB/AAxAB	142	76	218	139	75	214
		total	311	126	437	302	120	422
	**T052**×**T003 (n = 91)**	ABxAA/BB	179	83	262	131	66	197
		ABxAB	28	67	95	15	43	58
		BB/AAxAB	12	73	85	11	52	63
		total	219	223	442	157	161	318
	**T052**×**T064 (n = 123)**	ABxAA/BB	96	113	209	82	89	171
		ABxAB	137	130	267	132	111	243
		BB/AAxAB	97	138	235	89	121	210
		total	330	381	711	303	321	624
	**Unique**		**872**	**656**	**1528**	**829**	**569**	**1398**
***Malus***	**RG×GS (n = 186)**	ABxAA/BB	3	1020	1023			
		ABxAB	3	587	590			
		BB/AAxAB	7	1203	1210			
		total	13	2810	2823			

OH×LBJ = ‘Old Home’×‘Louise Bon Jersey’; T003×M = P128R068T003×‘Moonglow’; T042×T081) = P019R045T042×P037R048T081; T052×T003 = P202R137T052×P128R068T003; T052×T065 = P202R137T052×P266R225T064.

### Identification and Genotyping of SNPs with Null Alleles

**Figure 3:**
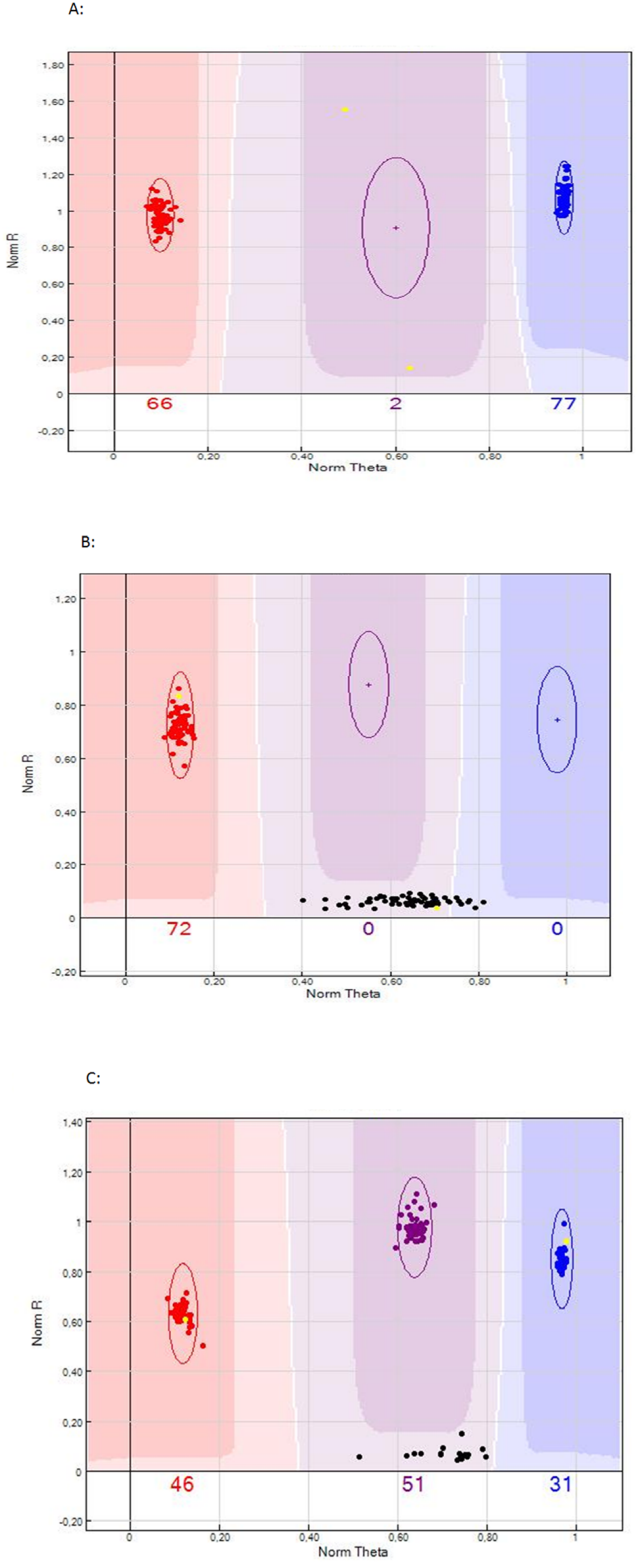
Typical examples of SNPs with null allele as represented in GenomeStudio. A) A 00×AB SNP (ss527789894), as represented in GenomeStudio. Parents P128R068T003 and ‘Moonglow’ are indicated in yellow; the red and blue clusters are identified as A0 and B0 genotypes, respectively. The total number of the individuals analyzed is 143 and the segregation ratio is 1∶1. B) A 00×A0 SNP (ss475879014), as represented in GenomeStudio. Parents P128R068T003 and ‘Moonglow’ are indicated in yellow; the red cluster is identified as heterozygous genotypes (A0), while genotypes with missing call (in black) are identified as homozygous for the null allele (00). The total number of the individuals analyzed is 143 and the segregation ratio is 1∶1. C) A A0×B0 SNP (ss475882353), as represented in GenomeStudio. Parents P128R068T003 and ‘Moonglow’ are indicated in yellow; the red, blue and purple clusters are identified as A0, B0 and AB genotypes, respectively, while genotypes with missing call (in black) are identified as homozygous for the null allele (00). The total number of the individuals analyzed is 143 and the segregation ratio is 1∶1:1∶1.

The analysis of SNP polymorphism in segregating populations highlighted the presence of SNP markers with potential null alleles. By default, the standard SNP calling algorithms of GenomeStudio clustered heterozygous A0 and B0 genotypes together with homozygous AA and BB genotypes, and called homozygous null genotypes (00) as missing genotypic calls. However, some SNPs containing null alleles do not follow the expected Mendelian segregation based on the parental genotypes. Therefore, manual editing of clusters for all the SNPs with strong deviation from Mendelian ratio or around 25% or 50% of no calls was performed and the SNPs which displayed a clear clustering and for which genotypes could be unequivocally determined as containing potential null alleles, were selected for further linkage analysis (Figures 3A, B and C). The following null allele segregation types were observed in the segregating populations: 00×A0, A0×AA, A0×A0, A0×B0, AB×A0, A0×BB and AB×00. The number of polymorphic null allele SNPs varied throughout the five populations: 115 in OH×LBJ, 108 in T003×M, 112 in T042×T081, 702 in T052×T003, and 436 in T052×T064 (Table 2). The percentage of polymorphic null allele markers from attempted bead types seemed to be similar for pear and apple SNPs: 2% and 1.2% in OH×LBJ, 2.9% and 1% in T003×M, 2.4% and 1.1% in T042×T081, 9.9% and 8.1% in T052×T003, and 4.9% and 5% in T052×T064. Of the total of 1132 unique pear and apple SNPs exhibiting null alleles, 255 were polymorphic markers without a null allele in at least one other segregating population. When the polymorphic null allele markers were mapped, the null allele markers were used to increase the density of the maps for the interspecific crosses, but were not required for the already dense OH×LBJ map (Table 3).

**Table 2:** Number of polymorphic and mapped null allele markers for each segregating population.

			Null- allele markers			Mapped null- allele markers		
	Population	Marker segregation	Pear SNPs	Apple SNPs	Total	Pear SNPs	Apple SNPs	Total
***Pyrus***	**OH×LBJ** * **(n = 297)**	00×A0/00×B0/BB×B0	1	45	47	1	39	40
		A0×A0/B0×B0	17	46	63	9	28	37
		AB×00	0	0	0	0	0	0
		A0×B0	4	0	7	3	0	3
		A0×AB/AB×B0/AB×A0	0	2	2	0	1	1
		total	22	93	115	13	68	81
	**T003**×**M (n = 220)**	00×A0/00×B0/BB×B0	3	57	60	3	51	54
		A0×A0/B0×B0	0	6	6	0	6	6
		AB×00	11	5	16	11	5	16
		A0×B0	0	2	2	0	2	2
		A0×AB/AB×B0/AB×A0	9	2	11	9	2	11
		A0×BB/B0×AA	9	4	13	9	4	13
		Total	32	76	108	32	70	102
	**T042**×**T081 (n = 142)**	00×A0/00×B0/BB×B0	3	63	66	3	57	60
		A0×A0/B0×B0	9	20	29	9	20	29
		AB×00	1	0	1	0	0	0
		A0×AB/AB×B0/AB×A0	2	1	3	1	1	2
		A0×BB/BB×A0	11	2	13	10	1	11
		total	26	86	112	23	79	102
	**T052**×**T003 (n = 91)**	00×A0/00×B0/BB×B0	30	193	223	24	123	147
		A0×A0/B0×B0	40	421	461	10	76	86
		A0×B0	5	7	12	3	2	5
		A0×AB/B0×AB/AB×B0	1	5	6	2	3	5
		Total	76	626	702	39	204	243
	**T052**×**T064 (n = 123)**	00×A0/00×B0/BB×B0	32	213	245	18	134	152
		A0×A0/B0×B0	12	156	168	13	169	182
		A0×AB	4	1	5	2	1	3
		A0×B0	6	12	18	3	6	9
		Total	54	382	436	36	310	346
	**Unique**		**163**	**969**	**1132**	**117**	**557**	**674**

The number is shown for apple and pear SNPs separately, and in total. OH×LBJ = ‘Old Home’×‘Louise Bon Jersey’; T003×M = P128R068T003×‘Moonglow’; T042×T081) = P019R045T042×P037R048T081; T052×T003 = P202R137T052×P128R068T003; T052×T065 = P202R137T052×P266R225T064.

* null allele not used for mapping.

**Table 3:** Common mapped polymorphic SNP markers in each parent of the different segregating populations: diagonal in bold, total number of mapped markers in a specified parent (including null alleles); above the diagonal, null alleles; below the diagonal, polymorphic markers without null alleles.

		OH×LBJ		T003×M		T042×T081		T052×T003		T052×T064	
		OH	LBJ	T003	M	T042	T081	T052	T003	T052	T064
**OH×LBJ**	**OH**	**356** *	NA	NA	NA	NA	NA	NA	NA	NA	NA
	**LBJ**	104	**393** *	NA	NA	NA	NA	NA	NA	NA	NA
**T003**×**m**	**T003**	8	11	**182**	18	6	20	4	84	17	25
	**M**	105	130	13	**434**	76	52	52	12	51	48
**T042**×**T081**	**T042**	56	80	2	6	**250**	19	34	4	29	27
	**T081**	63	70	5	6	19	**312**	34	18	44	35
**T052**×**T003**	**T052**	32	50	8	10	4	2	**370**	58	40	50
	**T003**	10	12	20	14	6	3	6	**255**	27	43
**T052**×**T064**	**T052**	31	48	6	6	4	6	164	27	**628**	125
	**T064**	37	52	11	14	7	7	90	52	215	**682**

OH**×**LBJ = ‘Old Home’×‘Louise Bon Jersey’; T003×M = P128R068T003×‘Moonglow’; T042×T081) = P019R045T042×P037R048T081; T052×T003 = P202R137T052×P128R068T003; T052×T065 = P202R137T052×P266R225T064.

* no null alleles mapped.

The total number of unique polymorphic markers, including both apple and pear-derived SNPs and SNPs with null alleles, was 2400 for all five populations. For the pear SNPs, 918 (83.8%) were polymorphic in at least one segregating population, and 623 (56.8%) were polymorphic in OH×LBJ, 384 (35%) in T052×T064, 337 (30.7%) in T042×T081, 337 (30.7%) in T003×M, and 295 (26.9%) in T052×T003.

### Genetic Map Construction

Parental genetic maps were constructed for five segregating populations using the 2400 unique polymorphic SNPs. All maps contained 17 linkage groups except T003, T042 and T081(Table S3). For the OH×LBJ population, the parental maps spanned 825 and 974 cM and consisted of 356 and 393 SNP markers for OH and LBJ, respectively. For the T003×M population, the parental maps spanned 980 and 1016 cM and consisted of 182 and 434 SNP markers for T003 and M, respectively. For the T042×T081 population, the parental maps spanned 923 and 1133 cM and consisted of 250 and 312 SNP markers for T042 and T081, respectively. For the T052×T003 population, the parental maps spanned 1018 and 1101 cM and consisted of 370 and 255 SNP markers for T052 and T003, respectively. For T052×T064 the parental maps spanned 1485 and 1580 cM and consisted of 628 and 682 SNP markers for T052 and T064, respectively. In total, 1888 unique SNPs were mapped, including null allele markers.

**Figure 4:**
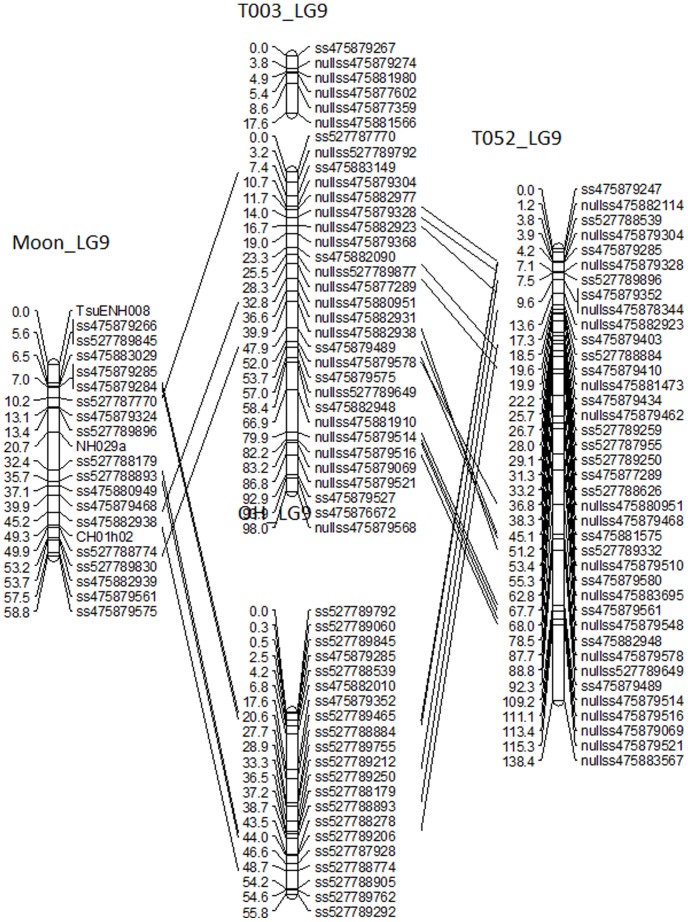
Alignment of LG9 from four parental maps P128R068T003, ‘Moonglow’, P202R137T052 and ‘Old Home’. The lines between the maps each show markers in common with two other parents.

The markers in common among the five segregating populations enabled the alignment of parental genetic maps as shown in Figure 4 for four maps of LG9. However, the bridges among the 10 parental maps were insufficient for the construction of a unique integrated map. The common polymorphic markers (with and without null alleles) between pairs of parents of the segregating populations are shown in Table 3. For example, there are 105 common polymorphic markers (without null alleles) between the European pears ‘Moonglow’ and ‘Old Home’. In comparison, only 52 markers (without null alleles) are in common between ‘Moonglow’ and the interspecific parent T081. The parent T003 from the T003×M cross has 20 null allele markers in common with the same parent from the T052×T003 cross and only 5 with T081.

### SSR Mapping

Of the 54 SSR markers derived from the published ‘Bartlett’ consensus map [24] that were screened over the T003×M population, 38 were mapped, 25 loci to T003 and 30 to ‘Moonglow’ (Table S1). This information on linkage group assignment, taken together with data on SNP markers in common, was sufficient to enable the application of the ‘Bartlett’ LG nomenclature across all the pear genetic maps in this study.

### Pear SNP Alignment to the Apple Genome Sequence

**Figure 5:**
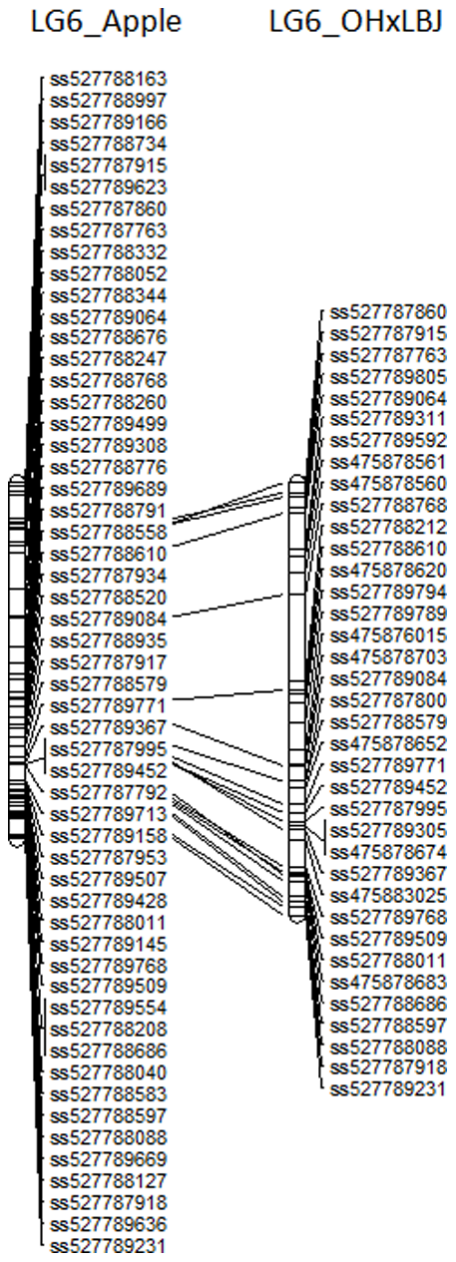
Alignment of OH×LBJ LG6 with chromosome 6 of the 'Golden Delicious' genome. Lines show the markers in common.

A total of 1009 pear SNPs (92%) were successfully anchored to the GD genome using bioinformatics analysis. Using the OH**×**LBJ consensus map as an example, 433 (42.9%) of the pear SNPs were anchored to apple and enabled the comparison of this genetic map with the GD genome assembly. On average, 20 markers per LG were in common between the OH**×**LBJ map and the GD genome (Figure 5), with LG2 having the most markers in common (32 markers) and LG17 the least (9 markers).

## Discussion

SNPs are considered to be the most efficient tools for comprehensive genetic studies [32]. In *Pyrus*, the number of available SNPs was marginal. We developed more than 1,000 SNPs from the re-sequencing of *P. communis* cultivars and for the first time we included them in an array, making them easily available for further studies. These SNPs were selected based on their location within candidate genes, to ensure their usefulness for marker-trait association and for future breeding programmes.

We used the apple and pear Infinium® II 9K SNP array for the genotyping of five segregating pear populations, for a grand total of 873 individuals. The clustering of the SNPs using the GenomeStudio software depends on the minor allele frequency of the SNPs: the lower the minor allele frequency, the more samples are required to achieve accurate representation of all clusters. Illumina recommends a population of 100 or more. In our case, all the populations had largely more than 100 individuals (except for T052×T003, with 91 progenies), and this large dataset of 873 individuals ensured an accurate clustering of array SNPs. Moreover, the threshold of 15% for the MAF is relatively high, in comparison with other studies using the same technique [33].

### High Polymorphism Rate for the Newly Developed Pear SNPs

A large proportion (83.8%) of the 1096 pear SNPs used to construct the first pear genotyping array were polymorphic in at least one segregating population, and 857 of these unique polymorphic pear markers (93.4%) were demonstrated to be useful for construction of genetic maps, using five populations of a range of genetic backgrounds across *P. communis*, *P. pyrifolia* and *P. bretschneideri*. These maps are the first dense SNP-based genetic maps for pear of any species. The previously developed maps in *Pyrus*, including those of Yamamoto et al. and Celton et al. [21], [22], [24], as well as an earlier map using pear SNPs constructed in ‘Bartlett’ and ‘Hosui’ [34], are not sufficiently dense to be useful for QTL analysis. Although Wu et al. [8] reported the development of 2005 SNPs in the course of anchoring the *P. bretschneideri* genome sequence, these SNPs are not available as a genotyping array, as they were obtained using genotyping by sequencing. In addition to the new *P. communis* pear SNPs developed in this study, we found that 1482 SNP markers derived from apple (19.3% of the total apple SNPs on the IRSC array) were polymorphic in pear, and 1031 of them were positioned on the pear genetic maps. The apple SNPs considerably improved the density of all maps, in some cases, e.g. T052×T003 and T052×T064, even doubling the number of mapped markers. In fact, because of the lower polymorphism of pear SNPs in the interspecific hybrid parents compared with the *P. communis* parents, the apple SNPs were necessary to saturate these maps.

The higher number of polymorphic pear markers identified in the European pear cross OH×LBJ compared with the four populations with an Asian pear background is because sequence data from OH and LBJ were used to design the pear SNPs, which also validates the bioinformatic SNP detection method used. In the T003×M population, the number of polymorphic pear SNPs in the European parent (‘Moonglow’) was significantly higher than in the hybrid (T003), again because the SNPs were derived from sequencing of *P. communis* accessions. However, the number of pear SNPs that were polymorphic in the interspecific parents was more variable, and reflects both the number of SNPs that are conserved between European and Asian pear and those that were introgressed from the European parent into the interspecific hybrid parents. The transferability of SNPs between species of the same genus has been reported previously in a few studies. These include the plant genera *Vitis* [35], *Citrus* [19] and *Eucalyptus* [36], as well as the mammalian genus *Bubalus* [37]. It is noteworthy that the transferability of SNPs between species was as high in these studies as observed in this study in *Pyrus*.

### SNP Transferability between Genera Pyrus and Malus

The distinguishing feature of the apple and pear Infinium® II 9K SNP array is its combination of SNPs from both *Malus* and *Pyrus*, making it the first cross-genera SNP array created. It therefore enables, for one of the first time, the assessment of SNP marker transferability between genera. Most of the numerous studies on genetic marker transferability in recent years have focused on SSR markers, including those concerning apple and pear [22], [25], [38], [39]. Previous attempts to transfer SNPs between genera involved a few accessions only of the non-targeted species, including the study of Micheletti et al. [40], who estimated the rate of transferability of the heterozygous state from *M.*×*domestica* to *P. communis* and *P. pyrifolia* using 237 apple SNPs. In the present study, we observed that 7562 apple SNPs (98.3%) were either monomorphic or polymorphic in at least one pear population, while only 130 did not hybridize well in all of them. The high percentage of hybridization of pear genomic DNA to apple SNPs and *vice versa* obtained in the present study are not surprising, given that *Malus* and *Pyrus* are closely related genera and might be expected to share high sequence similarity. Furthermore, both the pear and apple SNPs included in the array were selected to be located in coding genes, with the consequence that the flanking sequences are more likely to be conserved between species. Although many of the apple SNPs were monomorphic (but still hybridized to pear DNA) and were not useful for genetic mapping in the five pear populations, we were able to map 99 apple markers in the OH×LBJ population, 255 in T003xMoonglow, 199 in T042×T081, 365 in T052×T003, and 631 in T052×T064.

### SNPs with Null Alleles

The existence of null or unexpected alleles has been already demonstrated in several other SNP genotyping studies. Such alleles can be explained as deletions spanning a polymorphic site, secondary polymorphisms, or tri-allelic sites at the primary polymorphism [19], [41]. Since the SNP genotyping technology we used was the Infinium® II from Illumina, any putative third allele of polymorphic SNPs was not detectable and, therefore, in our study the SNPs with null alleles can fall only into the first two categories. Null alleles are an important source of polymorphisms; however, they are challenging to detect and analyze using SNP array software. In the present study, a higher number of SNPs with null alleles was detected in the interspecific populations than in the *P. communis* population. This was expected, as the frequency of null alleles increases with genetic distance between the samples genotyped and the discovery panel [19], because additional SNPs in the flanking sequence used for the Infinium® array design are more likely to occur between different species (Asian versus European pear) or genus (*Malus* versus *Pyrus*). We found that the within-species frequency of null alleles was similar in apple and pear SNPs. As heterozygous null alleles are useful for genetic mapping, we used them to increase map density in interspecific populations. It must be noted, however, that null alleles are a potential source of increased false positives in marker-trait association studies [42], [43].

### Pear and Apple Genome Synteny

In total, 92% of the pear SNPs included in the Infinium® II array were successfully anchored to the ‘Golden Delicious’ genome [5], and the alignment of the physical map with the OH×LBJ genetic map resulted in an average of 20 orthologous markers per LG. Nevertheless, the apple SNPs were not always located at the same position on the pear genetic map as in the apple genome, which, however, can also be explained by the finding that approximately 15% of the SNPs included in the 9 K array have been assigned erroneous positions on the ‘Golden Delicious’ reference sequence [33]. However, the number of orthologous markers between apple and pear identified in the present work (433 pear SNPs and 99 apple SNPs for OH×LBJ) is almost double the total found in previous studies (227). These studies included those by Pierantoni et al. [39], who demonstrated good genome colinearity between one apple and two pear genetic maps, using 41 and 31 mapped apple SSRs, respectively; Yamamoto et al. [38], who mapped apple and pear markers in European pear cultivars, and found that the position of 66 apple SSRs showed colinearity with the apple reference map; and Celton et al. [24], who aligned the genetic maps of two apple and pear cultivars constructed using apple and pear SSRs, and identified 90 colinear markers (53 pear and 37 apple SSRs) in common between the apple and pear genomes.

## Conclusions

We have thoroughly validated the apple and pear Infinium® II 9K SNP array, and demonstrated its usefulness for high throughput genotyping in breeding populations of *P. communis*, as well as those of a mixed genetic background that includes *P. communis*, *P. pyrifolia* and *P. bretschneideri*. Furthermore, we attested that the arrayed SNPs are transferable not only across these species, but also between the two closely related genera *Malus* and *Pyrus*.

The construction of high density gene-based genetic maps using our SNP array represents an important step for the discovery of chromosomal regions associated with commercially important horticultural traits, such as pest and disease resistance, orchard productivity and fruit quality [32] in pears derived from *P. communis*, *P. pyrifolia* and *P. bretschneideri*. The OH×LBJ population was a repeat of a cross [44] used to develop an understanding of genetic determinants of vigour control and precocity in pear rootstocks. The 400 seedlings planted in Motueka (New Zealand) are grafted with ‘Doyenné du Comice’ (*P. communis)* scions for the purpose of a QTL analysis of rootstock induced dwarfing in pear. The T003×M population was developed to study the genetic basis of resistance to pear scab (*Venturia pirina*), fire blight (*Erwinia amylovora*), pear psylla (*Cacopsylla pyri*) and pear sawfly (*Caliroa cerasi*). T003 (as most Asian pears in general) is not host to *V. pirina* [45], [46] and a good source of resistance to *C. pyri* and *C. cerasi* [47], while ‘Moonglow’ derives from fire blight-resistant cultivars ‘Roi Charles Würtenberg’ and ‘Seckel’. The T042×T081 population was created to develop an understanding of the genetic control of scab resistance in pear. We are using the T052×T003 and T052×T064 populations to investigate the genetic basis of a storage-related disorder “friction discolouration”, using genetic mapping in combination with metabolomic phenotyping to identify QTLs controlling the disorder. Such examples of applications of the apple and pear Infinium® II 9K SNP array demonstrate that it will produce a range of outcomes that can be applied to pear breeding programmes worldwide.

### Genomic Resources

The pear SNPs detected by sequencing, the pear SNPs chosen for the apple and pear Infinium® II 9K SNP array, and the GenomeStudio cluster file developed are deposited in the Genome Database for Rosaceae (www.rosaceae.org). SNPs are available in dbSNP (http://www.ncbi.nlm.nih.gov/projects/SNP/) under accessions ss527787751 to ss527789916.

## Supporting Information

Table S1 **List of SSR markers with primer sequence.** Segregation type and comparison of mapping position for WBC and T003×M on maps is also provided.
(XLSX)

Table S2 **List of 1096 pear SNPs on the pear Infinium® II 9K SNP array.** The NCBI dbSNP accession, location on the ‘Golden Delicious’ genome assembly is indicated.
(XLSX)

Table S3 **Genetic linkage maps of five populations used to validate the apple and pear 9K SNP array.**
(XLSX)
